# Comparative In Vitro Evaluation of Antibacterial and Osteogenic Activity of Polysaccharide and Flavonoid Fractions Isolated from the leaves of *Saussurea controversa*

**DOI:** 10.3390/molecules24203680

**Published:** 2019-10-12

**Authors:** Igor Khlusov, Elena Avdeeva, Valeria Shupletsova, Olga Khaziakhmatova, Larisa Litvinova, Ekaterina Porokhova, Yaroslav Reshetov, Irina Zvereva, Lyudmila Mushtovatova, Mariia Karpova, Artem Guryev, Irina Sukhodolo, Mikhail Belousov

**Affiliations:** 1 Research School of Chemistry & Applied Biomedical Sciences, Tomsk Polytechnic University, Tomsk 634050, Russia; khlusov63@mail.ru (I.K.); mvb63@mail.ru (M.B.); 2 Department of Morphology and General Pathology, Siberian State Medical University, Tomsk 634050, Russia; porohova_e@mail.ru (E.P.); staranie@mail.ru (I.S.); 3 Department of Pharmaceutical Analysis, Siberian State Medical University, Tomsk 634050, Russia; ferroplex2013@yandex.ru (Y.R.); titan-m@mail.ru (A.G.); 4 Basic Laboratory of Immunology and Cell Biotechnology, Immanuel Kant Baltic Federal University, Kaliningrad 236041, Russia; vshupletsova@mail.ru (V.S.); hazik36@mail.ru (O.K.); larisalitvinova@yandex.ru (L.L.); 5 Department of Microbiology and Virology, Siberian State Medical University, Tomsk 634050, Russia; zverevaif@mail.ru (I.Z.); mls2013@mail.ru (L.M.); karpova.mr@ssmu.ru (M.K.)

**Keywords:** *Saussurea controversa* DC (Asteraceae), polysaccharides, flavonoids, human adipose-derived multipotent mesenchymal stromal cells, cytotoxicity, alizarin red staining, *Staphylococcus aureus* growth

## Abstract

The osteogenic, cytotoxic, and antibacterial activities of polysaccharide (PS-SC) and flavonoid (F-SC) fractions isolated from the leaves extract of *Saussurea controversa* were studied in vitro. F-SC consists of the five quercetin glycosides in the ratio 2:8:10:1:4, which were isolated from the leaves extract of *S. controversa* and have been characterized previously. PS-SC was first isolated from the leaves extract of *S. controversa* and has been described. PS-SC consists in 30 compounds is characterized by a high degree of heterogeneity with a heterogeneity index of 19.74. The Mw and Mn of PS-SC were 108.6 and 5.5 kDa, respectively. Structural fragments are represented by galactose, arabinose, xylose, glucose, uronic acids, mannose, and rhamnose in a 10.1:3.3:2.2:2.1:1.7:0.9:0.5 molar ratio. F-SC as compared with PS-SC showed in vitro microbicidal (50 g/L) and better bacteriostatic (6.25 g/L versus 25 g/L of PS-SC) effects against the 24-h growth of *Staphylococcus aureus* strain 209 P and a 21-day absence of cytotoxicity on human adipose-derived multipotent mesenchymal stromal cells (hAMMSCs). Both fractions (PS-SC > F-SC) at doses of 10–50 mg/L stimulated differentiation of hAMMSCs into secreting osteoblasts accompanied by local mineralization of extracellular matrix. These fractions of *S. controversa* and especially F-SC, might be promising peroral drugs in the complex treatment of bone fractures and for prophylaxis of their infectious complications.

## 1. Introduction

Bacterial contamination is one of the main causes of periprosthetic joint infection (PJI) and implant failure [1]. The interface between an artificial surface and bone cells and tissue is a critical point of implant osteointegration/failure. Microorganisms impair close bone/device integration. With increasing incidence of untreatable osteomyelitis and PJI induced first of all by antibiotic-resistant bacteria [1] such as methicillin-resistant *Staphylococcus aureus*, plants and their extracts have again attracted much interest because of the potential to be substitutes for antibiotics in the complex therapy of diseases.

In this regard, a search for new osteogenic and antimicrobial agents to treat infectious complicated bone pathology is very relevant. There are numerous examples of clear evidence for a wide spectrum of in vitro antibacterial activity of plant fractions of flavonoids [2,3,4,5] and polysaccharides [6,7,8] at doses that are often comparable with reference antibiotics, including tetracycline, which is known to cure bone infections. The osteogenic potential of substances of natural origin is little known and of high interest in terms of their efficiency and safety.

An increase in osteoblastic activity and a decrease in osteoclastogenesis were found in vitro for the *Herba epimedii* extract containing flavonol glycosides [9]. This extract significantly decreased urinary calcium excretion, suppressed serum alkaline phosphatase (ALP) activity in ovariectomized rats and stimulated cell proliferation in vitro, increased ALP activity in osteoblast-like cells, modulated osteoclastogenesis by increasing osteoprotegrin mRNA and decreasing receptor activator of ligand RANKL mRNA expression. It was revealed in vitro that quercetin derivatives and other flavonoids effectively inhibit osteoclast versus osteoblast proliferation [10,11]. The osteoprotective efficiency of calcium chelate compounds, (-)-epigallocatechingallate, and chlorogenic acid was also revealed [12,13]. The stimulating effect of polysaccharides from *Gnaphalium affine* on the process of maturation of the extracellular collagen matrix, which is an important component for bone mineralization, was established [14]. Polysaccharides from *Polygonatum sibiricum* restrained the osteoporosis progression by promoting osteoblast formation and blocking osteoclastogenesis through Wnt/β-catenin signaling pathway [15].

*Saussurea controversa* DC is used in traditional medicine by the people of Siberia as an antimicrobial and anti-inflammatory agent, for the treatment of diseases of the musculoskeletal system. *S. controversa* is a perennial plant of the Asteraceae family, growing mainly in the forest-steppe zone, in meadow-steppe phytocenoses, and on rocky slopes in Siberia, Buryatia, and the Far East. We have previously described some active substances isolated from *S. controversa* extract (native chelidonic acid and its complex with calcium) and their direct in vitro effect on osteogenic differentiation of human multipotent mesenchymal stromal cells (MMSCs) [16] promoting bone regeneration. Osteoprotective in vivo action of *S. controversa* extract, containing flavonoids and polysaccharides as predominant components, has been recently discovered in a rat model of experimental osteomyelitis caused by *Staphylococcus aureus* [17]. Flavonoids of *S. controversa* decreased the intensity of inflammatory processes and stimulated bone regeneration as compared with the antibiotic cefazolin [18].

Hence, it was of current interest to do a comparative in vitro study of the predominant osteogenic activity or antibacterial effect against a pathogenic strain of *Staphylococcus aureus* induced by the polysaccharide and flavonoid fractions of *S. controversa* extract.

## 2. Results

### 2.1. The Structure of Components

The experiment used a total of five flavonol glycosides (F-SC), which were isolated from the leaves extract of *S. controversa* in the ratio 2:8:10:1:4 and have been characterized previously (quercetin 7-*O*-α-l-rhamnopyranoside-3-*O*-β-d-glucopyranoside; quercetin3-*O*-β-d-glucopyranosyl-(1→6)-β-d-glucopyranosyl-(1→6)-*O*-α-l-rhamnopyranoside; quercetin 3-*O*-β-d-glucopyranosyl-(1→6)-*O*-α-l-rhamnopyranoside; quercetin 7-*O*-α-l-rhamnopyranoside-3-*O*-β-d-xylopyranoside; quercetin 7-*O*-β-d-glucopyranoside-3-*O*-α-l-rhamnopyranosyl-(1→2)-*O*-β-d-glucopyranoside) [10].

Polysaccharides from *S. controversa* extract have not been previously isolated and described. The yield of purified and lyophilized PS-SC was 4.8% of the extract. PS-SC includes polysaccharides with different molecular weights. It contains 21 polysaccharides with a high molecular weight of over 5 kDa (5.6 to 304.9 kDa), it content was 65.9% of the PS-SC fraction. In addition, PS-SC contains 9 compounds with a molecular weight below 5 kDa (0.9 to 4.8 kDa), it content was 34.1% of the PS-SC fraction. The protein content in PS-SC was 16.15 ± 0.48%. The weight-averaged molecular weight (Mw) of PS-SC was 108.6 and number-averaged molecular weight (Mn) was 5.5 kDa. PS-SC is characterized by a high degree of heterogeneity; the index of heterogeneity was 19.74. The dominant structural components of PS-SC, determined by gas-liquid chromatography of alditol acetates, are galactose (40.53%) and arabinose (13.16%). Residues of xylose (8.95%), glucose (8.55%), mannose (3.65%), and rhamnose (2.16%) are minor components of the carbohydrate chains of PS-SC. The content of uronic acids was 6.71 ± 0.25%. Thus, PS-SC is a galactoarabinan with a Mw of 108.6 and comprised galactose, arabinose, xylose, glucose, uronic acids, mannose, and rhamnose in a 10.1:3.3:2.2:2.1:1.7:0.9:0.5 molar ratio.

### 2.2. Study of Cytotoxicity and Osteogenic Differentiation of hAMMSCs In Vitro

The data of the in vitro investigation demonstrated different effects of F-SC (an absence of cytotoxicity) and PS-SC on the number of viable human adipose-derived multipotent mesenchymal stromal cells (hAMMSCs) after a long-term 21-day cultivation in a standard nutrient medium. PS-SC reduced the number of viable stromal stem cells by 4–8 times in comparison with a 21-day control culture of hAMMSCs at all tested doses (10–50 mg/L) (Table 1). However, the amount of dead (apoptotic and necrotic) cells did not differ statistically from the control level and corresponding values of F-SC. Strong limitation of hAMMSC culture biomass growth was proposed to promote enhanced osteogenic differentiation.

**Figure 1:**
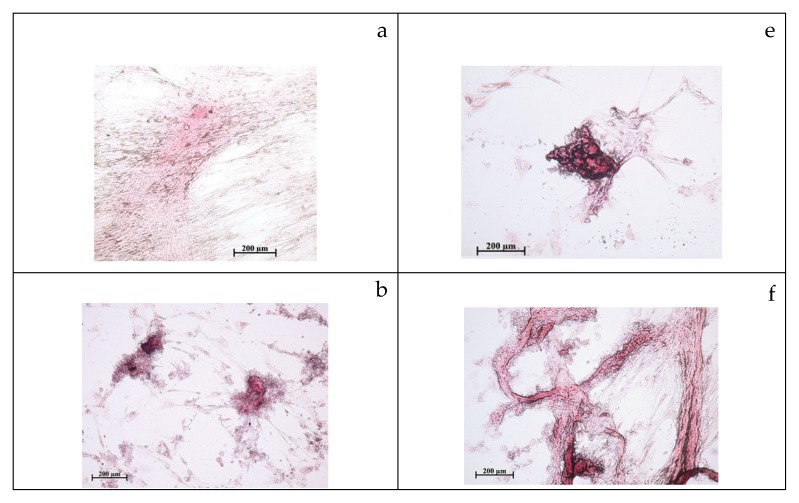

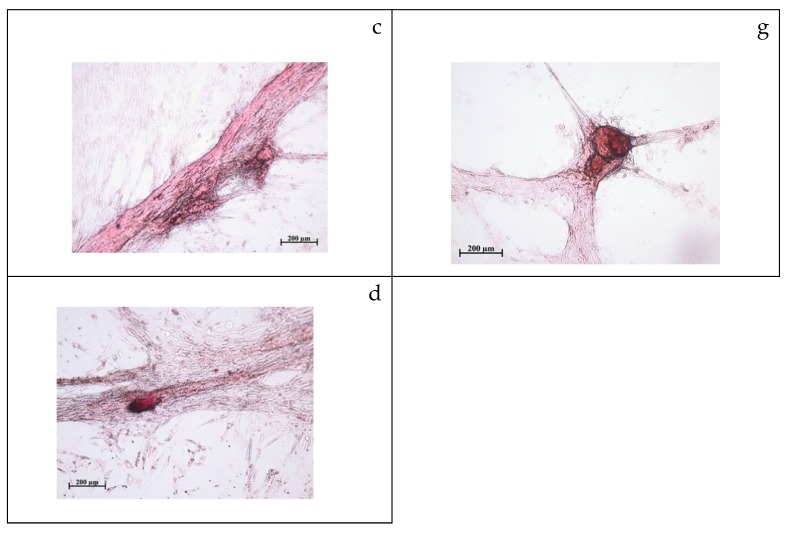
Culture of human adipose-derived multipotent mesenchymal stromal cells after 21 days of cultivation in a standard nutrient medium α-MEM with the addition of the different doses of tested compounds: a—control; b—10 mg/L of F-SC; c—30 mg/L of F-SC; d—50 mg/L of F-SC; e—10 mg/L of PS-SC; f—30 mg/L of PS-SC; g—50 mg/L of PS-SC. Staining with alizarin red S.

Indeed, both fractions (mainly PS-SC) dose-dependently increased the number (up to 9 times) and optical density (up to 7 times) of alizarin red stained sites in cell cultures. The average areas of the mineralization sites corresponded to the control level (Table 2). Therefore, stimulation of three-dimensional (3D) locations with predominant hAMMSC differentiation into osteoblasts (Figure 1) without massive cell proliferation (PS-SC > F-SC) was proposed for tested fractions of *S. controversa* extract.

### 2.3. Study of Bacterial Cell Growth In Vitro

Significant *S. aureus* growth occurred on a nutrient agar medium while 0.9% NaCl solvent was mixed with a microbial suspension at a 1:1 ratio. The area of bacterial CFUs observed in Petri dishes was 45% (Table 3). When F-SC or PS-SC dilutions were introduced into the suspension, the area of CFU growth decreased statistically by 9–11% if the doses of tested fractions of *S. controversa* reached 6.25 and 25 g/L, respectively (Table 3).

**Figure 2:**
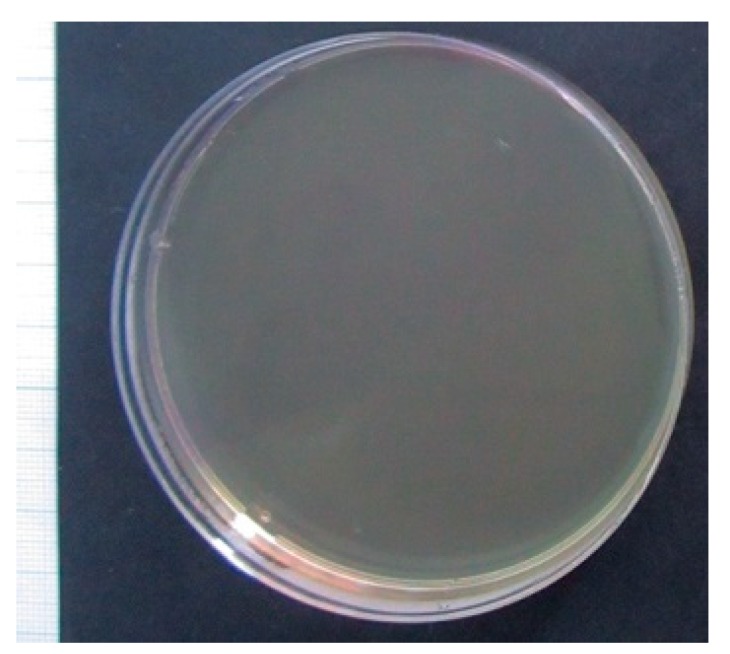
Growth of *S. aureus* strain 209P inhibited for 24 h after spending 24 h in a preliminary mixture with F-SC at a dose of 50 g/L.

Moreover, it was established that growth of *S. aureus* strain 209P was fully inhibited for 24 h after being in a preliminary mixture with F-SC at a dose of 50 g/L, which was attributed to microbicidal influence (Figure 2). No microbicidal effect was shown for any concentrations (0.781–100 g/L) of PS-SC.

## 3. Discussion

*S. controversa* extract contains two times more polysaccharides than flavonol glycosides. Flavonol glycosides have a hydrophilic character and are represented by di- and tri-glycosides of quercetin at the 3 and 7 positions of the flavone nucleus. Their sugar components are mainly rhamnose and glucose, as well as xylose. Polysaccharide fractions from the *S. controversa* extract have the structure of a galactoarabinan. It consists of galactose, arabinose, xylose, glucose, uronic acids, mannose, and rhamnose in a 10.1:3.3:2.2:2.1:1.7:0.9:0.5 molar ratio.

Many natural polysaccharides are characterized by their biocompatibility, low toxicity, biodegradable properties [19], and anti-osteoporotic effects [20]. According to Yu et al. (2019) [7], the extracts of *Ramulus mori* polysaccharides (RMPs) contained seven monosaccharides, namely, mannose, rhamnose, glucuronic acid, glucose, xylose, galactose, and arabinose, in a 1.36:2.68:0.46:328.17:1.53:21.80:6.16 molar ratio and exhibited cytotoxic effects on RAW 264.7 mouse macrophage-like leukemia virus transformed cell line at a dose range of 5–10 g/L. It is necessary to emphasize the short-term 24–48 h investigation of RMPs cytotoxicity described by [7]. In turn, the dependence of cytotoxicity on incubation time is known [21]. Most likely, small concentrations of polysaccharides can decrease cell viability in the case of prolonged contact, as revealed in a 21-day culture of hAMMSCs at doses of 10–50 mg/L of PS-SC (Table 1).

At the same time, the number of dead (apoptotic and necrotic) stromal cells did not differ statistically from the control level (Table 1). Therefore, a limitation of viable hAMMSC growth but not cytotoxicity of PS-SC was proposed. A diminished proliferation rate of MSCs, which increased their ability to differentiate into the osteogenic lineage and to deposit mineralized matrix, was noted by Rodríguez et al. [22]. Accordingly, dose-dependent elevation of the number of cell culture mineralization sites and their optical density (in other words, three-dimensionality of osteogenesis regions) with simultaneously diminished average areas of the mineralization sites caused by PS-SC (Table 2) support a hypothesis of hAMMSC-enhanced differentiation. Conversely, osteogenic 4-oxo-4H-pyran-2.6-dicarboxylic acid (chelidonic acid), which has also previously been isolated from *S. controversa*, significantly stimulated the in vitro growth of hAMMSC population [18].

Further, the RMPs had in vitro antimicrobial activity against *E. coli* and *P. aeruginosa* while *S. aureus* possessed highest bacterial resistance at a dose range of 1–20 g/L [7] versus our results for PS-SC at comparable doses (Table 3).

Flavonoids are small molecules, whereas polysaccharides are high-molecular carbohydrates. However, the osteogenic action of these substances may be similar. Polysaccharides may enhance the expression of the ALP and matrix metallopeptidase 13 (collagenase 3) (*MMP13*) gene from the early stage of differentiation, leading to maturation and mineralization of the collagenous extracellular matrix (ECM) [16]. **Polysaccharides** from *Persimmon* (*Diospyros kaki* L.f.) leaves consisting of arabinose, galactose, glucose, mannose, and rhamnose have in vivo and in vitro anti-osteoporotic effects in a model of ovariectomy-induced **bone** loss via dose-dependent inhibition of osteoclast differentiation through down-regulated activator of nuclear factor-κB ligand (RANKL) [20].

Flavonoids, in turn, decreased the urinary calcium excretion, stimulated osteoblast proliferation, increased ALP activity in osteoblast-like cells, and inhibited osteoclastogenesis by increasing osteoprotegrin mRNA and decreasing receptor activator of ligand RANKL mRNA expression [11,12,13].

According to our data, both F-SC and, to a lesser extent, PS-SC increased the number and optical density of the areas of mineralization sites in the hAMMSC culture. At the same time, the average mineralization area induced by substances at a dose of 50 mg/L was 2⅔ times higher for F-SC than for PS-SC (Table 2). Nevertheless, the cumulative (if the numbers and areas of mineralization are considered simultaneously) osteogenic effect is obviously similar for tested fractions on hAMMSC differentiation estimated in vitro via ECM mineralization. The formation of new bone matrix involving the alizarin red technique of staining is the most common in vitro protocol to assess the osteogenic (osteoinduction) capacity of tested drugs and materials [21].

The carbohydrate forms amorphous ECM in the form of long-chained polysaccharides called glycosaminoglycans (GAGs) with a vast spectrum of biological activity. With the exception of hyaluronic acid (which is unique among the GAGs because it is not sulfated), all GAGs are covalently attached to a protein backbone to form proteoglycans which are integral membrane and extracellular matrix (ECM) proteins and are thus modulators of cell growth and differentiation via soluble and fiber molecules of receptor- and cytoskeleton-mediated intracellular signaling and pathways [23].

Flavonoids, first of all quercetin and its derivates, have shown significant modulation in the levels of hyaluronic acid in ECM [24] and prevention of ECM degradation by matrix metalloproteinases [25].

Fabre et al. (2015) demonstrated the capacity of vitamin C and flavonoids to form non-covalent associations within lipid bilayers close to the membrane/cytosol interface with significantly enhanced antioxidant activity in these complexes [26]. Ascorbic acid is one of the osteogenic supplements in synthetic nutrient mediums for MMSC culturing [27]. In addition, vitamin C is required for hydroxylation of the collagen propeptide. After the modifications, the procollagen chains align and form the triple helix [23].

It should be noted that most molecular (mainly antioxidant) effects of flavonoids were described for endothelial and cancer cells [28]. However, information about the mechanisms of their osteogenic influence established for hAMMSCs (Table 2) is highly insufficient. Regulation of ECM stiffness may be one of the common targets of F-SC and PS-SC to regulate MMSC behavior through cytoskeleton-mediated mechanotransduction of molecules (i.e., Rho-family GTPases) and genes related to proliferation or differentiation (e.g., c-fos, egr-1, iex-1, c-myc) [29].

Despite very similar hAMMSC differentiation into osteoblasts enhanced in vitro by PS-SC and F-SC (Table 2), their differences in cytotoxicity (Table 1) and antibacterial activity (Table 3) were established. Flavonoids consisting of quercetin, isorhamnetin 3-*O*-b-d-xyloside, isorhamnetin 3-*O*-b-d-glucoside, quercetin 3-*O*-b-d-glucoside, quercetin 7-*O*-b-d-glucoside, and patuletin 3-*O*-b-d-glucoside were not toxic, and their LD_50_ values were greater than 80 mg/L [30]. F-SC also had no impact on stem cell viability (Table 1), and all tested doses possessed a cytoprotective influence in comparison with corresponding concentrations of PS-SC (Table 1). Our data accord with known cytoprotective effects of flavonoids on endothelial cells [31].

**Figure 3:**
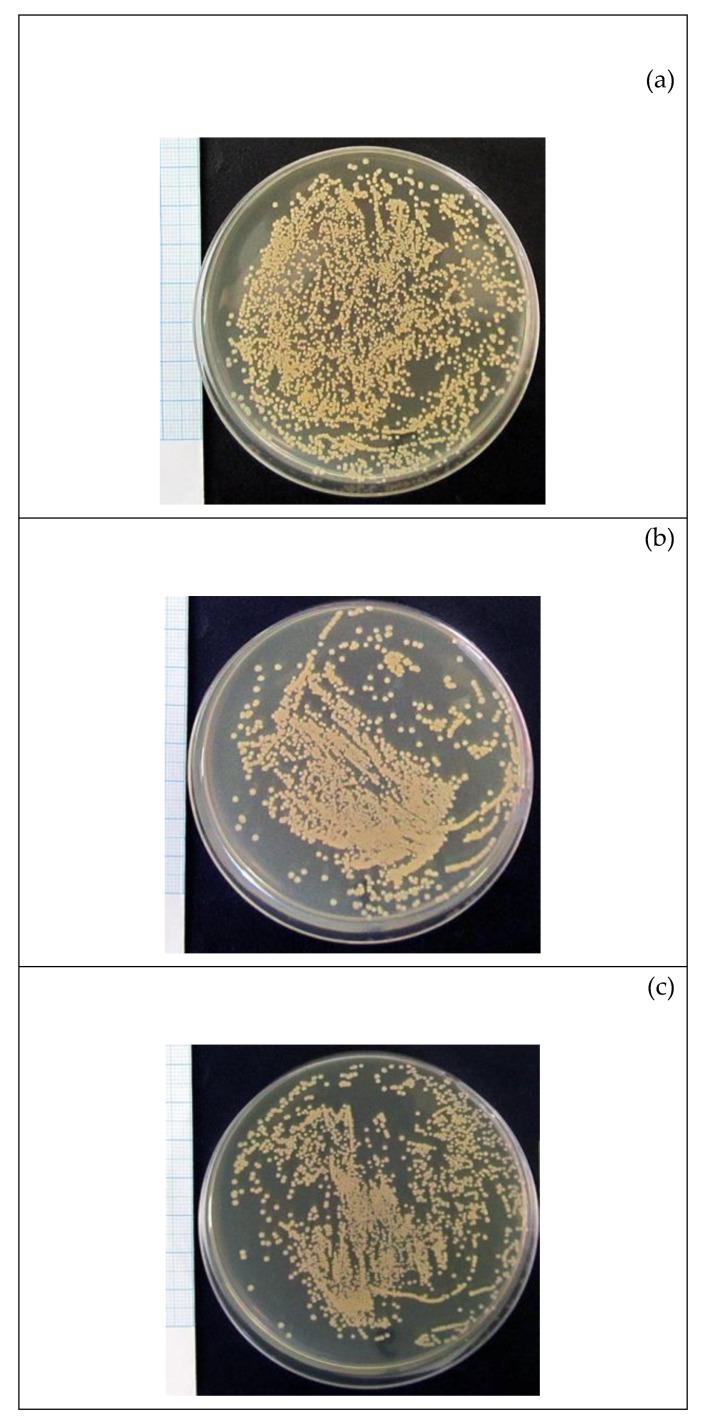
Results of growth of *S. aureus* strain 209P for 24 h after spending 24 h in a preliminary mixture with: (**a**) 0.9% NaCl solution (control); (**b**) F-SC; (**c**) PS-SC at a dose of 6.25 and 25 g/L, respectively.

Furthermore, the bacteriostatic dose of F-SC (6.25 g/L) against *S. aureus* in vitro growth was four times less than the dose of PS-SC (Table 3, Figure 3). Similar antibacterial doses of compositions extracted from leaves, peel, and flesh of *White guava* (*Psidium guajava* L. cv. Pearl) against *S. aureus* and *E. coli* reached 5 g/L [32], which corresponds to our results. 50 g/L of F-SC had a microbicidal effect (Figure 2). Similar microbicidal influence of PS-SC at a dose range of 0.781–100 g/L was not revealed.

Some accessible references displayed the highest activity of various flavonoids (flavon, flavonol, flavonones, flavane 3-ol, chalkone) and their compounds against *S. aureus* growth with the lowest concentrations of compounds 40–80 mg/L [30] or 0.25–625 mg/L [3,5] including methicillin-resistant *S. aureus* [4]. The proposed antibacterial mechanisms of flavonoids are as follows: inhibition of nucleic acid synthesis, inhibition of cytoplasmic membrane function, inhibition of energy metabolism, inhibition of the attachment and biofilm formation, inhibition of the porin on the cell membrane, alteration of the membrane permeability, and attenuation of the bacterial pathogenicity [4]. Excluding some of them, there are apparently similar pathways of mammalian cytotoxicity. Therefore, comparable doses of in vitro cytotoxicity against mainly mammalian tumor cell lines (Hep-G2, MCF-7, Jurkat T-cell leukemia) have been determined [33,34,35].

The specificity of the structure of flavonoids, in particular, of flavonols, determines their unique chemical properties and a wide range of biological activity. The presence of the flavone nucleus, oxo- and hydroxyl groups of phenolic nature cause the antioxidant, complexing, and weak acidic properties of flavonols. And the number and nature of the substituents add nuances in biological activity.

Thus, the 5.7-hydroxyl group is an indicator of antibacterial activity for flavones and flavonols, and additional hydroxyl groups at position 3 and in the phenolic ring (4^/^) significantly increase this type of activity [4]. Glycosidization of hydroxyl groups to positions 3 and 7 in F-SC increases the hydrophilicity of the molecule and is more likely to reduce antibacterial activity in vitro, since it has been discovered that almost all hydrophobic substituents (aliphatic chain, heterocyclic fragments et al.) enhance this type of activity [4]. At the same time, the antibacterial activity of F-SC in vivo should be significantly higher after hydrolysis of glycosides as a result of their metabolism, and glycosidization of flavonols reduces toxicity and gives advantages in bioavailability [36].

Not only the basic structure of the flavone nucleus and the antioxidant and complexing properties of phenolic groups in the ring have a role in the osteogenic activity of F-SC, but also the number and nature of the bonds of sugar fragments [12,13]. The latter assumption is confirmed by the revealed osteogenic properties of polysaccharides, mainly consisting of sugar monoses and having different types of glycoside chains [16,17,37]. The composition of sugar fragments plays a decisive role in the activity of polysaccharides, and the degree of polymerization of the branch and the degree of branching determine the degree of activity. Structural and physico-chemical properties are also the main factors affecting the biological activity of polysaccharides [37].

**Figure 4:**
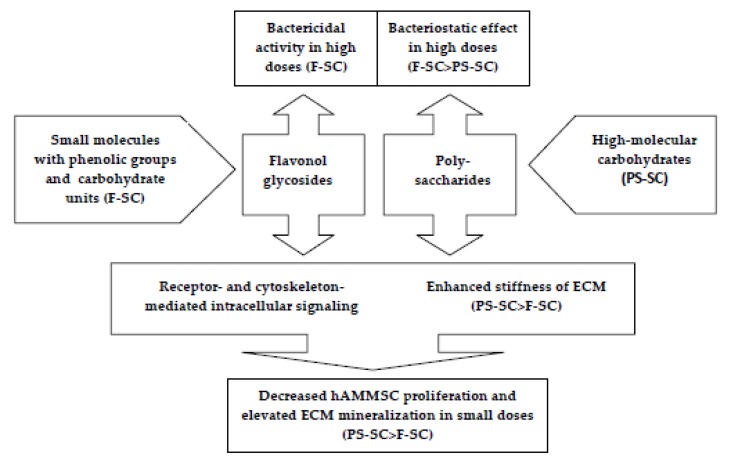
Speculative scheme of common and different pathways of F-SC and PS-SC in vitro activities.

Comparative accumulation of possible in vitro mechanisms of F-SC and PS-SC antibacterial and osteogenic effects was represented in Figure 4.

In our experiments, F-SC had no long-term limiting influence on healthy hAMMSC growth, unlike PS-SC, and both substances promoted in vitro osteogenic differentiation of cells at concentrations many times less (125–2500 times) as compared with their antibacterial activities. Thus, the direct effect on osteogenic differentiation of MMSCs has to take precedence over antimicrobial properties to promote bone restoration in conditions of experimental osteomyelitis caused by *S. aureus*, which we described previously for *S. controversa* compounds at a dose range of 10–100 mg/kg [9,10].

## 4. Materials and Methods

### 4.1. Plant Material

Leaves of *S. controversa* were collected in the region of Irkutsk, Russia, during the flowering phase in July 2013 and were air-dried. The plants were collected by Prof. A. A. Semenov and identified by Prof. M. N. Shurupova. A voucher specimen (No. TK-004605) has been deposited at the Herbarium of Tomsk State University (Tomsk, Russia).

### 4.2. Extraction and Isolation

Raw materials (600 g) were extracted with hot 40% EtOH (3 × 6000 mL, 80°, 1 h each). The extract was filtered and evaporated until it became an aqueous residue, which was treated sequentially in a separating funnel with CHCl_3_ (3 × 200 mL) and ethyl acetate (6 × 200 mL) to remove lipophilic components. Then flavonoids were extracted from the water residue using *n*-butanol (10 × 200 mL). When *n*-butanol was removed, a butanol fraction with a yield of 13.00 g (2.2%) was obtained. The isolation and determination of structure of five flavonol glycosides (F-SC), which are major components of the butanol fraction, have been previously described [10].

The water residue obtained after the removal of n-butanol was concentrated under vacuum to 0.5 L, added drop by drop to the 2 L of 96% ethanol for the precipitation of polysaccharides, and kept for 12 h at room temperature. The resulting precipitate of polysaccharides was filtered on a Buchner funnel and washed with 95% ethanol. The isolated sum of polysaccharides was dissolved in water and extracted with a mixture of chloroform-isoamylol 5:1 (*v*/*v*) in a ratio of 1:5 (*v*/*v*) for 0.5 h. Then the aqueous layer containing polysaccharides was centrifuged and separated. The purified polysaccharide solution was placed into a semipermeable membrane (with pore diameter of 15 kDa), and was dialyzed against purified water for 30 h. The resulting dialysate was lyophilized (PS-SC).

### 4.3. Polysaccharides Study

The protein content (%) was determined by a UNICO 2800 spectrophotometer (United Products and Instruments, Wichita, KS, USA) by the Lowry method with preliminary protein precipitation by trichloracetic acid. The calculation was carried out on the basis of a calibration schedule obtained for standard solution of bovine serum albumin [38]. The quantitative content of uronic acids (%) was determined by the reaction of carbohydrate oxidation products with 3.5-dimethylphenol in the presence of concentrated sulfuric acid on a UNICO 2800 spectrophotometer (United Products and Instruments, USA) [39]. The content of uronic acids (%) was calculated according to the calibration schedule obtained for D-galacturonic acid in the concentration range 0.05–10 mg/mL.

Characteristics of polysaccharides molecular weight were determined by high performance exclusion chromatography in comparison with solutions of standard dextran samples (1 mg/mL) with Mw 15, 40, 60, 90, 110, 250, and 500 kDa (Sigma-Aldrich, Darmstadt, Germany) [40]. The index of heterogeneity (Mw/Mn) was calculated as the ratio of the weight-averaged molecular weight (Mw) to the number-averaged molecular weight (Mn) of polysaccharides. The content of high- and low-molecular polysaccharides in the sample was calculated by adding the peak area of substances relative to the molecular weight of 5000 Da.

The monosaccharide composition was determined after acid hydrolysis of polysaccharides with a 2-M solution of trifluoroacetic acid and subsequent acetylation of the resulting alditol monosis. Myoinositol was used as an internal standard. Identification of alditol acetates of monosaccharides was carried out on a Varian 450-GC chromatograph (Varian, Palo Alto, CA, USA) equipped with a flame ionization detector using a VF-5 MS capillary column (Varian, USA; 0.25 mm, 30 m); the analysis was carried out in a temperature regime from 175 °C (1 min) to 250 °C (2 min) at a temperature increase rate of 3 °C/min. The content of monosaccharides (%) was calculated from the peak area relative to the internal standard using the simple normalization method.

### 4.4. Human Cell Culturing and In Vitro Staining

The experiment was done as described earlier [18]. Adult human adipose-derived multipotent mesenchymal stromal cells (hAMMSCs) were isolated from lipoaspirates of healthy volunteers (permission no. 7 from December 9, 2015; the Local Ethics Committee, Innovation Park, Immanuel Kant Baltic Federal University) as described in [41]. The cells were stained using a Phenotyping Kit, human (130-095-198) and Viability Fixable Dyes (MiltenyiBiotec, Bergisch Gladbach, Germany), and the results were analyzed with a MACS Quant flow cytometer (MiltenyiBiotec, Bergisch-Gladbach, Germany) and KALUZA Analysis Software (Beckman Coulter, Brea, CA, USA) in accordance with the manufacturer’s instructions. More than 99% of the viable cells expressed CD73, CD90, or CD105 markers and did not display CD45, CD34, CD20, or CD14 markers (less than 1%). A 21-day culture of hAMMSCs showed the presence of osteoblasts, chondrocytes, and adipocytes [18]. This confirmed that AMMSCs belonged to the pool of mesenchymal stem cells according to the recommendations of the International Society for Cellular Therapy (ISCT) and the International Federation for Adipose Therapeutics and Science (IFATS) [42,43].

To determine the effect of F-SC and PS-SC on osteogenic (into osteoblasts) differentiation of hAMMSCs, cells were cultured in an incubator (Sanyo, Japan) at 100% humidity with 5% carbon dioxide at 37 °C for 21 days (the medium was replaced with fresh medium every 3–4 days). The cell suspension was prepared at an initial concentration of 5 × 10^4^ viable cells/mL in 1.5 mL of the following culture medium: 90% αMEM medium (Gibco Life Technologies; Grand Island, NY, USA), 10% fetal bovine serum (Sigma-Aldrich, St. Louis, MO, USA), 50 mg/L of gentamicin (Invitrogen, Carlsbad, CA, USA), and sterile L-glutamine solution freshly added to a final concentration of 280 mg/L (Sigma-Aldrich, USA). The compounds (final concentration 10, 30, or 50 mg/L) were added to the hAMMSC culture in plastic wells of a 24-well flat-bottom plate (Orange Scientific, Braine-l’Alleud, Belgium) initially and each time the medium was replaced.

Because the self-differentiation potency of hAMMSCs caused by the tested compounds could be established, the culture medium had no osteogenic supplements (β-glycerophosphate, dexamethasone and ascorbic acid). The wells were air-dried. The attached cells in the 21-day culture were fixed with 10% formalin for 1 h, washed with phosphate buffer, and stained with 2% alizarin red (Sigma-Aldrich, USA) to visualize the extracellular matrix. The osteoblasts with mineralization sites around them were stained in cell cultures with 2% alizarin red S as described above. Digital images of the hAMMSC culture were obtained with a Zeiss Axio Observer A1 microscope (Carl Zeiss Microscopy, LLC, Thornwood, NY, USA) as described in [18]. The numbers of sites and average areas of alizarin red staining (in mm^2^) were calculated in 3 wells for each dose of tested substances via quantitative computer histomorphometry with the help of ImageJ v. 1.43 software (National Institutes of Health, USA; http://www.rsb.info.nih.gov/ij). An optical density (OD) of the mineralizing sites was calculated with the help of Adobe Photoshop version 13.1.2 Software by means of their ability to absorb or reflect visible light according to the statistics of gray levels. The algorithm described in [44] was used.

Another 3 wells for each dose of tested substances were used to evaluate their possible cytotoxicity in 21 days of hAMMSC culturing. The cells were disaggregated with 0.05% trypsin (PanEco, Russia) in 0.53 mM EDTA (Sigma-Aldrich, USA), and the concentration of viable and dead (apoptotic, necrotic) cells was determined with a Countess™ Automated Cell Counter (Invitrogen, Carlsbad, CA, USA) using 0.4% trypan blue solution (Invitrogen, Carlsbad, CA, USA).

### 4.5. Bacterial Cell Growth In Vitro

An investigation was done with the pathogenic strain 209 P *of Staphylococcus aureus* (*S. aureus*) (the collection of the Department of Microbiology of Siberian State Medical University, Tomsk, Russia) as described earlier [9,10] with little modification. Strain 209P exhibits typical morphological, biochemical, hemolytic, and plasma-coagulating properties of *S. aureus*.

Microbial suspension (1000 microbial bodies) with F-SC, PS-SC, or pure solvent (0.9% NaCl solution as a control) was prepared in sterile conditions in 15-mL plastic tubes at a proportion of 1:1 (0.5:0.5 mL/mL) and was incubated for 24 h at 36.6 °C. End-point dilutions of 1/10 (100g/L), 1/20 (50g/L), 1/40 (25 g/L), 1/80 (12.5 g/L), 1/160 (6.25 g/L), 1/320 (3.125 g/L), 1/640 (1.563g/L), and 1/1280 (0.781g/L) were used for each studied fraction of *S. controversa*.

Then 0.2 mL of the suspension (100 microbial bodies) from each group was placed on a nutrient agar medium in 90 mm plastic Petri dishes and was cultured for 24 h at 36.6 °C and 100% humidity. Three Petri dishes were taken for each group. The method of computer morphometry was used (Image J v. 1.43 software, National Institutes of Health, USA) to calculate the areas of the bacterial colony forming units (CFUs; % of total area of Petri dish) because of the sites of microbial cell crowding.

### 4.6. Statistical Analysis

Statistical processing of results was carried out using the SPSS 17.0 statistical analysis software package. The following distribution parameters were calculated: the mean (X) and standard deviation (SD) or the median (Me), 25% (Q1) and 75% (Q3) quartiles. The normality of distribution was defined by a Kolmogorov–Smirnov test. Because of non-normal distribution, a non-parametric Mann–Whitney *U* test was used to evaluate the significant differences between the samples. Statistically significant differences were considered at a significance level of *p* < 0.05.

## 5. Conclusions

Two fractions of different chemical structures were isolated from the extract from *Saussurea controversa* leaves. The flavonoid fraction as compared with polysaccharides showed in vitro microbicidal and better bacteriostatic effects in the absence of cytotoxicity. Both fractions stimulated (PS-SC > F-SC) hAMMSC differentiation into secreting osteoblasts accompanied by nodular ECM mineralization (plaques of densely mineralized regions) as a known marker of calcium salt deposition in the late (terminal) stage of MMSC osteogenic differentiation [45]. 

Therefore, the polysaccharide and, to a greater extent, flavonoid fractions of *Saussurea controversa* extract might be prospective peroral drugs in the complex treatment of bone fractures and for prophylaxis of their infectious complications.

## Figures and Tables

**Table 1:** The indices of human adipose-derived multipotent mesenchymal stromal cells (hAMMSCs) viability after 21 days of culturing in the presence of tested compounds, Me (Q1–Q3).

Group, *n* = 3		Dose, mg/L	Number of Viable Cells, ×10^3^ cells/mL	Number of Apoptotic Cells, ×10^3^ cells/mL	Number of Necrotic Cells, ×10^3^ cells/mL
1	Stem cells cultured on plastic surface without compounds (control)	0	83 (48–91)	2.4 (0.4–3.0)	9.5 (2–14)
2	Cells + F–SC	10	88 (62–93)	6.7 (2.9–6.8)	26 (13–27)
		30	83 (67–89)	3.2 (2.1–3.5)	19.5 (11–25)
		50	70 (59–82)	2.6 (1.9–3.3)	12 (8–17)
3	Cells + PS–SC	10	15 (11–30) U_1_ < 0.05 U_3_ < 0.05	0.8 (0.3–1.6) U_3_ < 0.05	1.6 (1.5–5.2) U_3_ < 0.05
		30	21 (18–42) U_1_ < 0.05 U_3_ < 0.05	3 (2.1–3.8) U_2_ < 0.05	9 (6.3–13.7) U_2_ < 0.05
		50	11 (9–23) U_1_ < 0.05 U_3_ < 0.05	0.7 (0.2–1.8) U_2_ < 0.05 U_3_ < 0.05	1.5 (1.3–4.8) U_2_ < 0.05 U_3_ < 0.05

Statistical significances are shown according to the Mann-Whitney *U* test: U_1_—with the control; U_2_—with the doses of 30 and/or 10 mg/L; U_3_—with the corresponding doses of other substances; *n*—the number of tested wells of cultural plate for each dose.

**Table 2:** In vitro effect of tested compounds on hAMMSC osteogenic differentiation with mineralization of cell culture matrix, X ± SD, Me (Q1–Q3).

Group, *n* = 3		Dose, mg/L	The Number of Sites of Cell Culture Mineralization	*U* Criterion, Statistical Significance	Average Area of the Mineralization Sites, mm^2^	*U* Criterion, Statistical Significance	Optical Density (OD) of the Mineralization Sites, c.u.	*U* Criterion, Statistical Significance
1	Stem cells cultured on plastic surface without compound (control)	0	14 ± 2	-	0.027 (0.013–0.095)	-	8.10 (5.29–11.37)	-
2	Cells + F-SC	10	54 ± 5	U_1_ < 0.05	0.039 (0.019–0.093)	-	28.26 (23.73–36.05)	U_1_ < 0.05
		30	21 ± 4	U_1_ < 0.05 U_2_ < 0.05	0.029 (0.007–0.051)	-	32.87 (30.24–38.14)	U_1_ < 0.05
		50	53 ± 3	U_1_ < 0.05 U_2_ < 0.05	0.048 (0.020-0.083)	U_2_ < 0.05	41.81 (35.61–51.83)	U_1_ < 0.05
3	Cells + PS-SC	10	47 ± 3	U_1_ < 0.05	0.037 (0.011–0.179)	-	45.91 (35.47–55.51)	U_1_ < 0.05 U_3_ < 0.05
		30	118 ± 5	U_1_ < 0.05 U_2_ < 0.05 U_3_ < 0.05	0.025 (0.009–0.090)	-	48.72 (41.93–60.07)	U_1_ < 0.05 U_3_ < 0.05
		50	128 ± 13	U_1_ < 0.05 U_2_ < 0.05 U_3_ < 0.05	0.018 (0.009–0.064)	U_2_ < 0.05 U_3_ < 0.05	55.93 (44.10–67.33)	U_1_ < 0.05 U_3_ < 0.05

Statistical significances are shown according to the Mann-Whitney *U* test: U_1_—with the control; U_2_—with the doses of 30 and/or 10 mg/L; U_3_—with the corresponding doses of other substance; *n*—the number of tested wells of cultural plate for each dose; c.u.—conventional units of optical density.

**Table 3:** Minimal bacteriostatic doses of tested compounds suppressing growth of *S. aureus* strain 209P for 24 h in an agar medium after spending 24 h in a preliminary liquid culture, Me (Q1–Q3).

Group, *n* = 3		Dose, g/L	Area of the Bacterial Colonies, %	*U* Criterion, Statistical Significance
1	Control bacterial culture without compounds	0	45.2 (42.8–46.4)	-
2	*S. aureus* + F-SC	6.25	36.1 (35.4–36.7)	U_1_ < 0.05
3	*S. aureus* + PS-SC	25	34.2 (33.6–35.4)	U_1_ < 0.05

Statistical significances are shown according to the Mann-Whitney *U* test: U_1_—with the control; *n*—the number of tested Petri dishes studied in each group.
